# Crystal structure of 6-bromo-7-fluoro-4-oxo-4*H*-chromene-3-carbaldehyde

**DOI:** 10.1107/S2056989015011871

**Published:** 2015-06-27

**Authors:** Yoshinobu Ishikawa

**Affiliations:** a School of Pharmaceutical Sciences, University of Shizuoka, 52-1 Yada, Suruga-ku, Shizuoka 422-8526 , Japan

**Keywords:** crystal structure, chromone, hydrogen bonding, halogen inter­action

## Abstract

In the title compound, C_10_H_4_BrFO_3_, a brominated and fluorinated 3-formyl­chromone derivative, all atoms are essentially coplanar (r.m.s. deviation of 0.025 Å for the non-H atoms), with the largest deviation from the least-squares plane [0.050 (6) Å] being for a benzene-ring C atom. In the crystal, mol­ecules are linked through stacking inter­actions [centroid–centroid distance between the benzene and pyran rings = 3.912 (4) Å], C—H⋯O hydrogen bonds and short C⋯O contacts [2.865 (7) Å]. Unsymmetrical halogen⋯halogen inter­actions between the bromine and fluorine atoms [Br⋯F = 3.116 (4) Å, C—Br⋯F = 151.8 (2), C—F⋯Br = 154.1 (4)°] are also formed, giving a meandering two-dimensional network propagating in the (041) plane. A comparison with related structures is made and the various types of weak inter­actions are ranked in importance.

## Abstract 2

In the crystal of this brominated and fluorinated 3-formyl­chromone derivative, mol­ecules are linked through stacking inter­actions, C—H⋯O hydrogen bonds and short C⋯O contacts. Unsymmetrical halogen⋯halogen inter­actions between the bromine and fluorine atoms are also formed, giving a meandering two-dimensional network propagating in the (041) plane.

## Chemical context   

**Figure 1:**
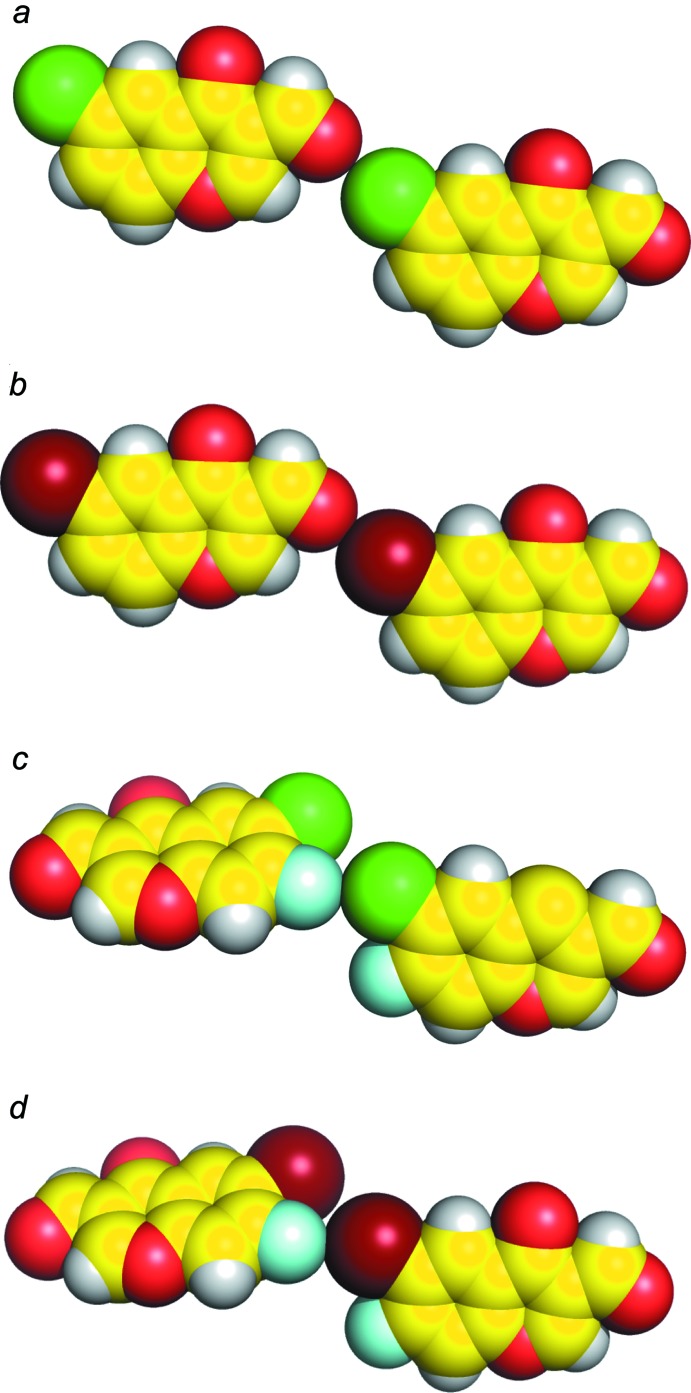
Sphere models of the crystal structures of (*a*) 6-chloro-4-oxo-4*H*-chromene-3-carbaldehyde (Ishikawa, 2014*a* ▸), (*b*) 6-bromo-4-oxo-4*H*-chromene-3-carbaldehyde (Ishikawa, 2014*b* ▸), (*c*) 6-chloro-7-fluoro-4-oxo-4*H*-chromene-3-carbaldehyde (Ishikawa, 2014*c* ▸) and (*d*) the title compound.

Halogen bonds and halogen⋯halogen inter­actions have recently attracted much attention in medicinal chemistry, chemical biology, supra­molecular chemistry and crystal engineering (Auffinger *et al.*, 2004 ▸; Metrangolo *et al.*, 2005 ▸; Wilcken *et al.*, 2013 ▸; Mukherjee & Desiraju, 2014 ▸; Metrangolo & Resnati, 2014 ▸; Persch *et al.*, 2015 ▸). I have recently reported the crystal structures of the halogenated 3-formyl­chromone derivatives 6-chloro-4-oxo-4*H*-chromene-3-carbaldehyde (Ishikawa, 2014*a* ▸), 6-bromo-4-oxo-4*H*-chromene-3-carbaldehyde (Ishikawa, 2014*b* ▸) and 6-chloro-7-fluoro-4-oxo-4*H*-chromene-3-carbaldehyde (Ishikawa, 2014*c* ▸). A van der Waals contact between the formyl oxygen atom and the chlorine atom in 6-chloro-4-oxo-4*H*-chromene-3-carbaldehyde (Fig. 1 ▸ *a*) and a shorter contact (halogen bonding) between the formyl oxygen atom and the bromine atom in 6-bromo-4-oxo-4*H*-chromene-3-carbaldehyde (Fig. 1 ▸ *b*) are observed. On the other hand, an unsymmetrical halogen⋯halogen inter­action is formed between the chlorine and fluorine atoms in 6-chloro-7-fluoro-4-oxo-4*H*-chromene-3-carbaldehyde (Fig. 1 ▸ *c*). As part of our inter­est in these types of chemical bonding, I herein report the crystal structure of a brominated and fluorinated 3-formyl­chromone derivative 6-bromo-7-fluoro-4-oxo-4*H*-chromene-3-carbaldehyde. The objective of this study is to reveal the inductive effect of the vicinal electron-withdrawing substit­uent on the bromine atom at the 6-position and the inter­action mode(*s*).
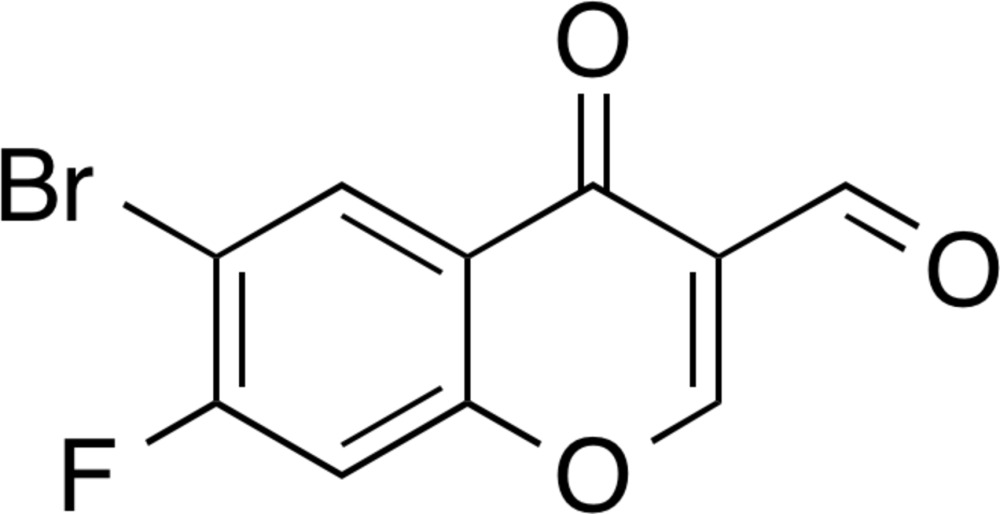


## Structural commentary   

**Figure 2:**
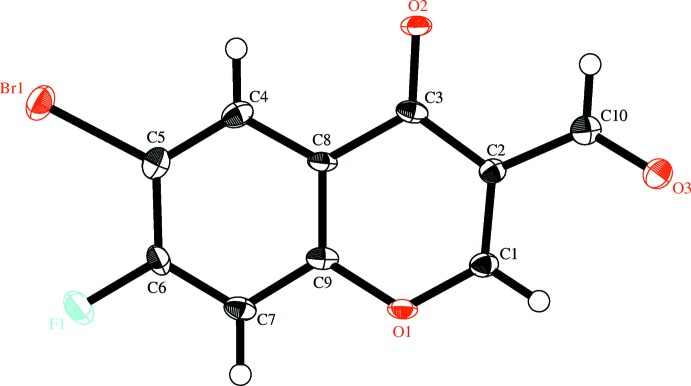
The mol­ecular structure of the title compound, with displacement ellipsoids drawn at the 50% probability level. Hydrogen atoms are shown as small spheres of arbitrary radius.

The title compound is shown in Fig. 2 ▸. The mean deviation of the least-square plane for the non-hydrogen atoms is 0.0253 Å, and the largest deviation is 0.050 (6) Å for C4. This means that these atoms are essentially coplanar.

## Supra­molecular features   

**Figure 3:**
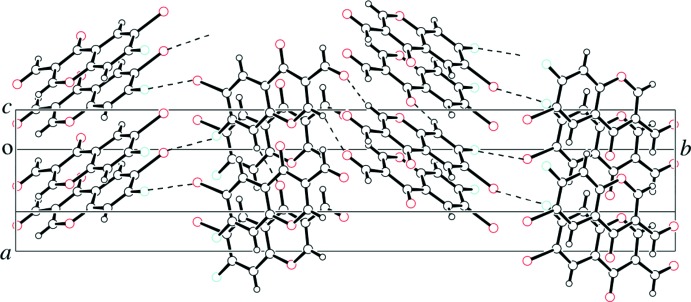
A packing view of the title compound. C—H⋯O hydrogen bonds and Br⋯F unsymmetrical halogen⋯halogen inter­actions are represented as dashed lines.

In the crystal, the mol­ecules are linked through stacking inter­actions between the translation-symmetry equivalent^i^ [centroid–centroid distance between the benzene and pyran rings of the 4*H*-chromene units = 3.872 (4) Å, symmetry code: (i) *x*, *y*, *z* − 1], and through C—H⋯O hydrogen bonds (Table 1 ▸), as shown in Fig. 3 ▸.

A contact between the formyl oxygen atom and the bromine atom is not found in the title compound. Instead, an unsymmetrical halogen⋯halogen inter­action is formed between the bromine and fluorine atoms [Br1⋯F1 = 3.116 (4) Å, C5—Br1⋯F1(−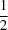 + *x*, 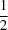 − *y*, 3 − *z*) = 151.8 (2)°, C6—F1⋯Br1(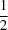 + *x*, 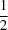 − *y*, 3 − z = 154.1 (4)°], as shown in Fig. 1 ▸ *d*. It is suggested that the electron-withdrawing fluorine atom at the 7-position should make the σ-hole of the bromine atom at the 6-position larger, and the electropositive region of the bromine atom should contact the electronegative region of the fluorine atom (Hathwar & Guru Row, 2011 ▸). Thus, halogen bonds (Cl⋯O and Br⋯O) are not observed in 6-chloro-7-fluoro-4-oxo-4*H*-chromene-3-carbaldehyde and the title compound, which might support the idea that the unsymmetrical halogen⋯halogen inter­actions (Cl⋯F and Br⋯F) are more favorable than the halogen bonds.

In addition to the C—H⋯O hydrogen bonds and the unsymmetrical halogen⋯halogen inter­action, a short contact between the formyl C10 and O3^ii^ atoms [2.865 (7) Å, (ii): –*x* + 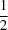, –*y*, *z* + 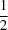, Fig. 3 ▸] is revealed in the title compound. This extraordinary inter­action is also observed in 6-chloro-7-fluoro-4-oxo-4*H*-chromene-3-carbaldehyde (Ishikawa, 2014*c* ▸), but is not observed in 6-chloro-4-oxo-4*H*-chromene-3-carbaldehyde (Ishikawa, 2014*a* ▸), 6-bromo-4-oxo-4*H*-chromene-3-carbaldehyde (Ishikawa 2014*b* ▸) and 7-fluoro-4-oxochromene-3-carbaldehyde (Asad *et al.*, 2011 ▸). Thus, this inter­esting feature might be caused by a strong dipole–dipole inter­action between the formyl groups polarized extremely by introducing both the bromine and fluorine atoms into the chromone ring. These findings should be helpful in the understanding of inter­actions of halogenated ligands with proteins, and thus invaluable for rational drug design.

## Synthesis and crystallization   

5-Bromo-4-fluoro-2-hy­droxy­aceto­phenone was prepared from 4-bromo-3-fluoro­phenol by Fries rearrangement reaction. To a solution of 5-bromo-4-fluoro-2-hy­droxy­aceto­phenone (7.56 mmol) in *N*,*N*-di­methyl­formamide (15 ml) was added dropwise POCl_3_ (18.9 mmol) at 273 K. After the mixture had been stirred for 14 h at room temperature, water (50 ml) was added. The precipitates were collected, washed with water, and dried *in vacuo* (yield: 74%). ^1^H NMR (400 MHz, CDCl_3_): *δ* = 7.33 (*d*, 1H, *J* = 8.0 Hz), 8.52 (*s*, 1H), 8.54 (*s*, 1H), 10.36 (*s*, 1H). Colorless plates were obtained by slow evaporation of a 1,2-di­meth­oxy­ethane/*n*-hexane solution of the title compound at room temperature.

## Refinement   

Crystal data, data collection and structure refinement details are summarized in Table 2 ▸. The C*sp* ^2^-bound hydrogen atoms were placed in geometrical positions [C–H 0.95 Å, *U*_iso_(H) = 1.2*U*_eq_(C)], and refined using a riding model.

## Supplementary Material

Crystal structure: contains datablock(s) General, I. DOI: 10.1107/S2056989015011871/hb7440sup1.cif

Structure factors: contains datablock(s) I. DOI: 10.1107/S2056989015011871/hb7440Isup2.hkl

CCDC reference: 1407902

Additional supporting information:  crystallographic information; 3D view; checkCIF report

## Figures and Tables

**Table 1:** Hydrogen-bond geometry (, )

*D*H*A*	*D*H	H*A*	*D* *A*	*D*H*A*
C1H1O3^i^	0.95	2.41	3.240(7)	146
C7H3O2^ii^	0.95	2.26	3.166(7)	158

Symmetry codes: (i) 
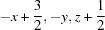
; (ii) 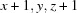.

**Table 2:** Experimental details

Crystal data	
Chemical formula	C_10_H_4_BrFO_3_
*M*_r_	271.04
Crystal system, space group	Orthorhombic, *P*2_1_2_1_2_1_
Temperature (K)	100
*a*, *b*, *c* ()	5.784(3), 33.713(14), 4.633(3)
*V* (^3^)	903.4(8)
*Z*	4
Radiation type	Mo *K*
(mm^1^)	4.56
Crystal size (mm)	0.30 0.25 0.10

Data collection	
Diffractometer	Rigaku AFC7R diffractometer
Absorption correction	scan (North *et al.*, 1968 ▸)
*T*_min_, *T*_max_	0.388, 0.634
No. of measured, independent and observed [*F* ^2^ > 2.0(*F* ^2^)] reflections	1744, 1384, 1203
*R*_int_	0.033
(sin /)_max_ (^1^)	0.650

Refinement	
*R*[*F* ^2^ > 2(*F* ^2^)], *wR*(*F* ^2^), *S*	0.046, 0.123, 1.12
No. of reflections	1384
No. of parameters	136
H-atom treatment	H-atom parameters constrained
_max_, _min_ (e ^3^)	1.21, 1.53
Absolute structure	Flack (1983 ▸), 110 Friedel pairs
Absolute structure parameter	0.02(3)

Computer programs: *WinAFC* (Rigaku, 1999 ▸), *SIR2008* (Burla *et al.*, 2007 ▸), *SHELXL97* (Sheldrick, 2008 ▸), *CrystalStructure* (Rigaku, 2010 ▸).

## supplementary crystallographic information

### Crystal data

**Table:**

C_10_H_4_BrFO_3_	*F*(000) = 528.00
*M**_r_* = 271.04	*D*_x_ = 1.993 Mg m^−^^3^
Orthorhombic, *P*2_1_2_1_2_1_	Mo *K*α radiation, λ = 0.71069 Å
Hall symbol: P 2ac 2ab	Cell parameters from 25 reflections
*a* = 5.784 (3) Å	θ = 15.1–16.6°
*b* = 33.713 (14) Å	µ = 4.56 mm^−^^1^
*c* = 4.633 (3) Å	*T* = 100 K
*V* = 903.4 (8) Å^3^	Plate, colorless
*Z* = 4	0.30 × 0.25 × 0.10 mm

### Data collection

**Table:**

Rigaku AFC7R diffractometer	*R*_int_ = 0.033
ω scans	θ_max_ = 27.5°
Absorption correction: ψ scan (North *et al.*, 1968)	*h* = −7→4
*T*_min_ = 0.388, *T*_max_ = 0.634	*k* = 0→43
1744 measured reflections	*l* = −3→6
1384 independent reflections	3 standard reflections every 150 reflections
1203 reflections with *F*^2^ > 2.0σ(*F*^2^)	intensity decay: −3.0%

### Refinement

**Table:**

Refinement on *F*^2^	Hydrogen site location: inferred from neighbouring sites
*R*[*F*^2^ > 2σ(*F*^2^)] = 0.046	H-atom parameters constrained
*wR*(*F*^2^) = 0.123	*w* = 1/[σ^2^(*F*_o_^2^) + (0.0881*P*)^2^] where *P* = (*F*_o_^2^ + 2*F*_c_^2^)/3
*S* = 1.12	(Δ/σ)_max_ < 0.001
1384 reflections	Δρ_max_ = 1.21 e Å^−^^3^
136 parameters	Δρ_min_ = −1.53 e Å^−^^3^
0 restraints	Absolute structure: Flack (1983), 110 Friedel Pairs
Primary atom site location: structure-invariant direct methods	Absolute structure parameter: 0.02 (3)
Secondary atom site location: difference Fourier map	

### Special details

**Table:**

Refinement. Refinement was performed using all reflections. The weighted *R*-factor (*wR*) and goodness of fit (*S*) are based on *F*^2^. *R*-factor (gt) are based on *F*. The threshold expression of *F*^2^ > 2.0 *σ*(*F*^2^) is used only for calculating *R*-factor (gt).

### Fractional atomic coordinates and isotropic or equivalent isotropic displacement parameters (Å^2^)

**Table:**

	*x*	*y*	*z*	*U*_iso_*/*U*_eq_	
Br1	0.12276 (11)	0.224324 (15)	1.22910 (13)	0.0241 (2)	
F1	0.5835 (7)	0.19539 (10)	1.4414 (8)	0.0246 (8)	
O1	0.6802 (7)	0.08180 (12)	0.8763 (9)	0.0163 (9)	
O2	0.0653 (7)	0.09910 (11)	0.4588 (9)	0.0181 (9)	
O3	0.4556 (7)	0.00248 (11)	0.2458 (10)	0.0206 (9)	
C1	0.6066 (10)	0.05663 (16)	0.6730 (12)	0.0156 (11)	
C2	0.4022 (10)	0.06057 (15)	0.5311 (12)	0.0136 (11)	
C3	0.2522 (10)	0.09394 (16)	0.5850 (12)	0.0146 (12)	
C4	0.2095 (10)	0.15532 (16)	0.8912 (13)	0.0174 (12)	
C5	0.2924 (11)	0.17998 (16)	1.1017 (14)	0.0174 (12)	
C6	0.5054 (10)	0.17161 (15)	1.2299 (14)	0.0174 (11)	
C7	0.6356 (10)	0.13944 (16)	1.1538 (13)	0.0166 (11)	
C8	0.3361 (9)	0.12153 (16)	0.8105 (12)	0.0143 (12)	
C9	0.5490 (10)	0.11445 (16)	0.9405 (12)	0.0142 (12)	
C10	0.3392 (10)	0.03094 (16)	0.3123 (12)	0.0173 (12)	
H1	0.7026	0.0347	0.6250	0.0187*	
H2	0.0664	0.1611	0.7995	0.0209*	
H3	0.7803	0.1343	1.2435	0.0199*	
H4	0.1959	0.0344	0.2153	0.0208*	

### Atomic displacement parameters (Å^2^)

**Table:**

	*U*^11^	*U*^22^	*U*^33^	*U*^12^	*U*^13^	*U*^23^
Br1	0.0215 (4)	0.0219 (3)	0.0289 (4)	0.0043 (3)	0.0054 (3)	−0.0050 (3)
F1	0.0229 (19)	0.0293 (18)	0.0217 (17)	−0.0055 (16)	−0.0022 (18)	−0.0089 (15)
O1	0.0088 (19)	0.024 (2)	0.0159 (19)	0.0017 (16)	−0.0026 (17)	−0.0016 (17)
O2	0.012 (2)	0.024 (2)	0.018 (2)	0.0042 (17)	−0.0052 (18)	−0.0009 (17)
O3	0.0170 (19)	0.0243 (19)	0.020 (2)	0.0020 (16)	0.002 (2)	−0.0038 (19)
C1	0.012 (3)	0.021 (3)	0.014 (3)	0.002 (3)	0.001 (3)	0.000 (2)
C2	0.014 (3)	0.016 (3)	0.011 (3)	0.002 (3)	0.000 (3)	0.0019 (19)
C3	0.010 (3)	0.022 (3)	0.012 (3)	−0.003 (3)	0.000 (3)	0.003 (3)
C4	0.014 (3)	0.021 (3)	0.018 (3)	0.003 (3)	0.002 (3)	0.003 (3)
C5	0.019 (3)	0.015 (3)	0.018 (3)	0.000 (3)	0.005 (3)	0.001 (3)
C6	0.020 (3)	0.016 (3)	0.016 (3)	−0.003 (3)	−0.001 (3)	−0.005 (3)
C7	0.011 (3)	0.024 (3)	0.015 (3)	−0.003 (3)	−0.001 (3)	0.001 (2)
C8	0.009 (3)	0.023 (3)	0.011 (3)	0.001 (2)	−0.002 (3)	0.001 (2)
C9	0.010 (3)	0.023 (3)	0.010 (3)	−0.001 (2)	0.001 (2)	0.005 (3)
C10	0.016 (3)	0.023 (3)	0.013 (3)	0.001 (3)	0.004 (3)	0.002 (3)

### Geometric parameters (Å, º)

**Table:**

Br1—C5	1.883 (6)	C4—C5	1.369 (9)
F1—C6	1.344 (7)	C4—C8	1.405 (8)
O1—C1	1.338 (7)	C5—C6	1.396 (9)
O1—C9	1.370 (7)	C6—C7	1.367 (8)
O2—C3	1.241 (7)	C7—C9	1.392 (8)
O3—C10	1.212 (7)	C8—C9	1.392 (8)
C1—C2	1.359 (8)	C1—H1	0.950
C2—C3	1.443 (8)	C4—H2	0.950
C2—C10	1.469 (8)	C7—H3	0.950
C3—C8	1.480 (8)	C10—H4	0.950
			
Br1···F1	3.004 (4)	Br1···H2	2.9352
F1···C9	3.588 (7)	F1···H3	2.5252
O1···C3	2.849 (7)	O1···H3	2.5222
O1···C6	3.588 (7)	O2···H2	2.6181
O2···C1	3.583 (7)	O2···H4	2.5704
O2···C4	2.881 (7)	O3···H1	2.5121
O2···C10	2.873 (7)	C1···H4	3.2714
O3···C1	2.831 (7)	C3···H1	3.2873
C1···C7	3.576 (8)	C3···H2	2.6949
C1···C8	2.764 (8)	C3···H4	2.6597
C2···C9	2.760 (8)	C5···H3	3.2809
C4···C7	2.800 (8)	C6···H2	3.2480
C5···C9	2.764 (8)	C8···H3	3.2878
C6···C8	2.754 (8)	C9···H1	3.1856
Br1···F1^i^	3.413 (4)	C9···H2	3.2692
Br1···F1^ii^	3.116 (4)	C10···H1	2.5561
F1···Br1^iii^	3.413 (4)	H1···H4	3.4920
F1···Br1^iv^	3.116 (4)	Br1···H2^v^	3.4115
F1···C4^v^	3.293 (7)	F1···H2^vi^	3.4488
F1···C5^v^	3.530 (8)	O1···H2^iii^	3.5011
F1···C8^v^	3.342 (7)	O1···H3^ix^	3.4733
F1···C9^v^	3.582 (7)	O1···H4^v^	3.5880
O1···O2^iii^	3.007 (6)	O2···H1^i^	3.1145
O1···O2^vi^	3.547 (6)	O2···H3^viii^	2.2632
O1···O3^v^	3.431 (6)	O3···H1^ix^	3.3905
O1···O3^vii^	3.589 (6)	O3···H1^x^	2.4077
O1···C2^v^	3.507 (7)	O3···H4^xi^	2.8893
O1···C3^iii^	3.596 (7)	O3···H4^xii^	2.6535
O1···C10^v^	3.303 (7)	C1···H3^ix^	3.4389
O2···O1^viii^	3.547 (6)	C1···H4^v^	3.5384
O2···O1^i^	3.007 (6)	C1···H4^xii^	3.5368
O2···C1^i^	3.174 (7)	C2···H3^ix^	3.5689
O2···C4^ix^	3.347 (8)	C2···H4^v^	3.5005
O2···C5^ix^	3.449 (7)	C2···H4^xii^	3.3605
O2···C7^viii^	3.166 (7)	C3···H3^viii^	3.4362
O2···C8^ix^	3.471 (7)	C3···H4^v^	3.5589
O3···O1^ix^	3.431 (6)	C4···H3^i^	3.0546
O3···O1^x^	3.589 (6)	C5···H2^v^	3.5448
O3···O3^xi^	3.324 (6)	C5···H3^i^	3.4028
O3···O3^xii^	3.324 (6)	C7···H2^iii^	3.0718
O3···C1^ix^	3.337 (7)	C8···H4^v^	3.5793
O3···C1^x^	3.240 (7)	C9···H2^iii^	3.4429
O3···C2^xi^	3.129 (7)	C9···H3^ix^	3.5587
O3···C3^xi^	3.545 (7)	C10···H1^x^	3.5611
O3···C10^xi^	2.865 (7)	C10···H4^xi^	3.5406
O3···C10^xii^	3.326 (7)	C10···H4^xii^	2.8936
C1···O2^iii^	3.174 (7)	H1···O2^iii^	3.1145
C1···O3^v^	3.337 (7)	H1···O3^v^	3.3905
C1···O3^vii^	3.240 (7)	H1···O3^vii^	2.4077
C1···C10^v^	3.452 (8)	H1···C10^vii^	3.5611
C2···O1^ix^	3.507 (7)	H1···H1^x^	3.3395
C2···O3^xii^	3.129 (7)	H1···H1^vii^	3.3395
C2···C7^ix^	3.456 (8)	H1···H4^iii^	3.4266
C2···C9^ix^	3.392 (8)	H1···H4^xii^	3.3038
C3···O1^i^	3.596 (7)	H2···Br1^ix^	3.4115
C3···O3^xii^	3.545 (7)	H2···F1^viii^	3.4488
C3···C6^ix^	3.422 (8)	H2···O1^i^	3.5011
C3···C7^ix^	3.356 (8)	H2···C5^ix^	3.5448
C3···C9^ix^	3.513 (8)	H2···C7^i^	3.0718
C4···F1^ix^	3.293 (7)	H2···C9^i^	3.4429
C4···O2^v^	3.347 (8)	H2···H3^viii^	3.1920
C4···C6^ix^	3.552 (9)	H2···H3^i^	2.7898
C4···C7^i^	3.576 (9)	H3···O1^v^	3.4733
C5···F1^ix^	3.530 (8)	H3···O2^vi^	2.2632
C5···O2^v^	3.449 (7)	H3···C1^v^	3.4389
C6···C3^v^	3.422 (8)	H3···C2^v^	3.5689
C6···C4^v^	3.552 (9)	H3···C3^vi^	3.4362
C6···C8^v^	3.323 (9)	H3···C4^iii^	3.0546
C7···O2^vi^	3.166 (7)	H3···C5^iii^	3.4028
C7···C2^v^	3.456 (8)	H3···C9^v^	3.5587
C7···C3^v^	3.356 (8)	H3···H2^iii^	2.7898
C7···C4^iii^	3.576 (9)	H3···H2^vi^	3.1920
C7···C8^v^	3.552 (8)	H4···O1^ix^	3.5880
C8···F1^ix^	3.342 (7)	H4···O3^xi^	2.6535
C8···O2^v^	3.471 (7)	H4···O3^xii^	2.8893
C8···C6^ix^	3.323 (9)	H4···C1^ix^	3.5384
C8···C7^ix^	3.552 (8)	H4···C1^xi^	3.5368
C9···F1^ix^	3.582 (7)	H4···C2^ix^	3.5005
C9···C2^v^	3.392 (8)	H4···C2^xi^	3.3605
C9···C3^v^	3.513 (8)	H4···C3^ix^	3.5589
C9···C10^v^	3.517 (8)	H4···C8^ix^	3.5793
C10···O1^ix^	3.303 (7)	H4···C10^xi^	2.8936
C10···O3^xi^	3.326 (7)	H4···C10^xii^	3.5406
C10···O3^xii^	2.865 (7)	H4···H1^i^	3.4266
C10···C1^ix^	3.452 (8)	H4···H1^xi^	3.3038
C10···C9^ix^	3.517 (8)	H4···H4^xi^	3.3354
C10···C10^xi^	3.284 (8)	H4···H4^xii^	3.3354
C10···C10^xii^	3.284 (8)		
			
C1—O1—C9	119.1 (5)	C3—C8—C4	121.8 (5)
O1—C1—C2	123.8 (5)	C3—C8—C9	119.2 (5)
C1—C2—C3	121.0 (5)	C4—C8—C9	119.0 (5)
C1—C2—C10	118.9 (5)	O1—C9—C7	116.2 (5)
C3—C2—C10	120.0 (5)	O1—C9—C8	122.3 (5)
O2—C3—C2	123.5 (5)	C7—C9—C8	121.5 (5)
O2—C3—C8	122.0 (5)	O3—C10—C2	125.2 (6)
C2—C3—C8	114.5 (5)	O1—C1—H1	118.124
C5—C4—C8	120.0 (6)	C2—C1—H1	118.120
Br1—C5—C4	121.5 (5)	C5—C4—H2	120.003
Br1—C5—C6	119.1 (5)	C8—C4—H2	120.030
C4—C5—C6	119.3 (6)	C6—C7—H3	121.140
F1—C6—C5	119.1 (5)	C9—C7—H3	121.139
F1—C6—C7	118.4 (5)	O3—C10—H4	117.421
C5—C6—C7	122.5 (6)	C2—C10—H4	117.409
C6—C7—C9	117.7 (6)		
			
C1—O1—C9—C7	−179.9 (4)	C8—C4—C5—Br1	−177.2 (5)
C1—O1—C9—C8	2.9 (8)	C8—C4—C5—C6	1.4 (9)
C9—O1—C1—C2	−2.7 (8)	H2—C4—C5—Br1	2.8
C9—O1—C1—H1	177.3	H2—C4—C5—C6	−178.6
O1—C1—C2—C3	2.5 (8)	H2—C4—C8—C3	−0.4
O1—C1—C2—C10	−179.8 (5)	H2—C4—C8—C9	177.8
H1—C1—C2—C3	−177.5	Br1—C5—C6—F1	0.2 (8)
H1—C1—C2—C10	0.2	Br1—C5—C6—C7	178.4 (4)
C1—C2—C3—O2	179.2 (5)	C4—C5—C6—F1	−178.4 (5)
C1—C2—C3—C8	−2.2 (8)	C4—C5—C6—C7	−0.2 (9)
C1—C2—C10—O3	0.3 (9)	F1—C6—C7—C9	178.1 (5)
C1—C2—C10—H4	−179.7	F1—C6—C7—H3	−1.9
C3—C2—C10—O3	178.1 (5)	C5—C6—C7—C9	−0.1 (9)
C3—C2—C10—H4	−1.9	C5—C6—C7—H3	179.9
C10—C2—C3—O2	1.4 (8)	C6—C7—C9—O1	−177.9 (5)
C10—C2—C3—C8	−180.0 (5)	C6—C7—C9—C8	−0.7 (8)
O2—C3—C8—C4	−0.8 (8)	H3—C7—C9—O1	2.1
O2—C3—C8—C9	−178.9 (5)	H3—C7—C9—C8	179.3
C2—C3—C8—C4	−179.4 (5)	C3—C8—C9—O1	−2.9 (8)
C2—C3—C8—C9	2.4 (7)	C3—C8—C9—C7	−179.9 (5)
C5—C4—C8—C3	179.6 (5)	C4—C8—C9—O1	178.9 (5)
C5—C4—C8—C9	−2.2 (8)	C4—C8—C9—C7	1.9 (8)

Symmetry codes: (i) *x*−1, *y*, *z*; (ii) *x*−1/2, −*y*+1/2, −*z*+3; (iii) *x*+1, *y*, *z*; (iv) *x*+1/2, −*y*+1/2, −*z*+3; (v) *x*, *y*, *z*+1; (vi) *x*+1, *y*, *z*+1; (vii) −*x*+3/2, −*y*, *z*+1/2; (viii) *x*−1, *y*, *z*−1; (ix) *x*, *y*, *z*−1; (x) −*x*+3/2, −*y*, *z*−1/2; (xi) −*x*+1/2, −*y*, *z*−1/2; (xii) −*x*+1/2, −*y*, *z*+1/2.

### Hydrogen-bond geometry (Å, º)

**Table:**

*D*—H···*A*	*D*—H	H···*A*	*D*···*A*	*D*—H···*A*
C1—H1···O3^vii^	0.95	2.41	3.240 (7)	146
C7—H3···O2^vi^	0.95	2.26	3.166 (7)	158

Symmetry codes: (vi) *x*+1, *y*, *z*+1; (vii) −*x*+3/2, −*y*, *z*+1/2.
